# *ACTB* Variants Confer the Genetic Susceptibility to Diabetic Kidney Disease in a Han Chinese Population

**DOI:** 10.3389/fgene.2019.00663

**Published:** 2019-07-23

**Authors:** Mengxia Li, Ming Wu, Yu Qin, Jinyi Zhou, Jian Su, Enchun Pan, Qin Zhang, Ning Zhang, Hongyan Sheng, Jiayi Dong, Ye Tong, Chong Shen

**Affiliations:** ^1^Department of Epidemiology, School of Public Health, Nanjing Medical University, Nanjing, China; ^2^Department of Non-communicable Chronic Disease Control, Jiangsu Provincial Center for Disease Control and Prevention, Nanjing, China; ^3^Department of Chronic Disease Prevention and Control, Huai’an City Center for Disease Control and Prevention, Huai’an, China; ^4^Changshu County Center for Disease Control and Prevention, Suzhou, China

**Keywords:** *ACTB* gene, single nucleotide polymorphisms, diabetic kidney disease, estimated glomerular filtration rate, type 2 diabetes mellitus

## Abstract

Beta-actin (ACTB) loss-of-function mutations result in a pleiotropic developmental disorder of kidney. The present study aims to explore whether the common variants at the *ACTB* gene contribute to diabetic kidney disease (DKD) susceptibility in patients with type 2 diabetes mellitus (T2DM). From the baseline population of 20,340 diabetic patients, 1,510 DKD cases and 1,510 age-matched T2DM controls were selected. All subjects were Han Chinese. Three tagging single nucleotide polymorphisms (SNPs), rs852423, rs852426, and rs2966449, at the *ACTB* gene were genotyped. Logistic regression was performed to estimate the association with DKD. SNPs, rs852426 and rs2966449, were significantly associated with DKD [additive model; odds ratio (OR), 1.217 and 1.151; *P* = 0.001 and 0.018, respectively]. The association of rs852426 with DKD still remained statistically significant after Bonferroni correction and particularly significant in the population older than 70 years rather than the 70 years or younger (*P* = 0.047 for heterogeneity test). Furthermore, the association of rs852426 with DKD was observed in populations of male and females without smoking, drinking, and with duration for T2DM 10–20 years. The association of rs2966449 with DKD was also found in the populations older than 70 years, male, not smoking, not drinking, and with duration for T2DM over 20 years. The estimated glomerular filtration rate (eGFR) levels of the individuals with TT or CC genotypes of rs2966449 were significantly lower than that of TC genotype in DKD cases (*P* = 0.021). The present study provides evidence that the *ACTB* variants, i.e., rs852426 and rs2966449, may confer the genetic susceptibility to DKD in a Han Chinese population.

## Introduction

According to the International Diabetes Federation (IDF) survey in 2017, approximately 451 million people aged 18 to 99 years worldwide are suffering from diabetes mellitus, and the number was expected to increase to 693 million by year 2045 (Cho et al., 2018). China Kadoorie Biobank (CKB) data from 170,287 participants showed that the estimated standardized prevalence of total diagnosed and undiagnosed diabetes in urban and rural China reached 10.9% in 2013 and that of pre-diabetic patients was 35.7% (Wang et al., 2017). In reality, diabetes mellitus and its complications seriously deteriorate patients’ quality of life (Trikkalinou et al., 2017).

Diabetic kidney disease (DKD), a well-established microvascular complications of diabetes mellitus, is also the most frequent primary cause of end-stage renal disease (ESRD) (Kdoqi, 2007). In 2014, the American Diabetes Association (ADA) and the National Kidney Foundation (NKF) defined DKD as a chronic kidney disease (CKD) caused by diabetes mellitus, which was characterized by glomerular basement membrane thickening, mesangial expansion, nodular sclerosis, and finally, advanced diabetic glomerulosclerosis (Tervaert et al., 2010). DKD accounted in more than 40% of ESRD (Remuzzi et al., 2002; Zelnick et al., 2017).

The all-age DKD mortality rates in China increased by 33.3% from 1990 to 2016 (Liu et al., 2018). Accordingly, the Global Burden of Disease (GBD) reported that deaths from DKD all over the world also significantly grew up with an increase percentage of 40.73% from 2007 to 2017 (Collaborators GBDCoD, 2018). Therefore, the growing prevalence and mortality due to DKD constitute a huge health and social economic burden and have become a serious public health problem in China and all over the world (Cooper, 2012).

DKD is a complex disease with multiple etiologies and pathogenesis, including genetic determinants, glucose/lipid metabolism disorders, glomerular hemodynamic changes, and abnormal expression of cytokines (Kong et al., 2013; Mauer et al., 2015; Kitada et al., 2016). Identification of genetic markers linked to DKD could provide deep insights into the underlying biological processes of the renal dysfunction. Previous studies suggested that variants in TGF-β/Smad classic pathway and Wnt/β-catenin signaling pathway were associated with DKD (Regele et al., 2015; Ying and Wu, 2017).

The *ACTB* gene encodes an abundant cytoskeletal housekeeping protein β-actin, which is ubiquitous in eukaryotic cells (Pollard and Cooper, 2009). Recently, a study suggests that a critically reduced amount of β-actin alters cell function and gene expression to the detriment of brain, heart, and kidney development (Cuvertino et al., 2017). *ACTB* loss-of-function mutations result in a pleiotropic developmental disorder, including unilateral renal agenesis, pelvic kidney, and kidney cysts, whereas there are few reports about the effect of the common variants at *ACTB* on the renal function. Although five genomewide association studies (GWAS) of DKD identified several susceptible loci (Iyengar et al., 2015; Lim et al., 2017; Gurung et al., 2018; van Zuydam et al., 2018; Ahluwalia et al., 2019; Guan et al., 2019), no near loci linked to *ACTB* were observed as well as the data from GWAS of the National Human Genome Research Institute (https://research.nhgri.nih.gov/).

This study aimed to evaluate the association between three tagging single-nucleotide polymorphisms (tagSNPs) in the *ACTB* gene and DKD in a Han Chinese cohort of T2DM patients. The findings would provide a better understanding of the genetic susceptibility of *ACTB* in DKD.

## Patients and Methods

### Study Participants

Subjects in this analysis were selected from the project of “Comprehensive Research on the Prevention and Control of the Diabetes (CRPCD),” which has been previously described in detail (Miao et al., 2017; Shen et al., 2018). According to the criteria proposed by the ADA in 2010 (American Diabetes Association, 2010), 19,992 subjects with type 2 diabetes mellitus (T2DM) were defined from all 20,340 participants. In the current study, we constructed a case-control study in T2DM subjects with equal DKD cases and controls. Referring to the recommendations released by NKF in 2007 (Kdoqi, 2007), a total of 3,443 T2DM patients who met one of the following three criteria would be selected as DKD cases: 1) eGFR less than 60 ml·min^−1^·1.73 m^−2^ (n = 2,193); 2) self-reported kidney disease (n = 810); 3) concomitant microvascular disease (mainly diabetic retinopathy) accompanied by CKD stage 2 (eGFR) less than 90 ml·min^−1^·1.73 m^−2^) (n = 1,663). Meanwhile, subjects with the following characteristics were excluded: 1) time interval between T2DM and DKD less than 2 years (n = 94); 2) non-DKD in mentioned self-reported kidney disease (n = 185); 3) diabetes mellitus duration less than 5 years (n = 1,281). Thus, 1,883 DKD cases could be included. We used propensity score matching (PSM) to match a non-DKD control, by age (2 years as an interval), for every case. Finally, 1,510 pairs were involved in this study. Informed consent was obtained from all participants and approved by the Research Ethics Committee of Jiangsu Provincial Center for Disease Control and Prevention and Nanjing Medical University.

### Questionnaire Survey and Anthropometric Measurements

All staff were uniformly trained and qualified in a standardized manner. Individuals accepted the survey by a questionnaire addressing their demographic characteristics, smoking, drinking, and self-reported chronic disease history. Anthropometric measurements including height, weight, waistline, and hipline were measured with light clothing. Body mass index (BMI) was then calculated as weight (kg)/height squared (m^2^).

### Definition of Drinking and Smoking

Drinking was defined as at least one alcoholic drink per month. Subjects who have ever smoked 100 cigarettes in the past were considered as smokers.

### SNP Selection

The *ACTB* gene maps to the chromosome 7p22.1 (Gene ID: 60; Locus NC_000007.14) and spans 3.4 kbp and consists of six exons. We searched the SNPs from the upstream 5 kb to the downstream 2 kb and selected tagSNPs through the database of the Chinese Han population in Beijing (CHB) and China of the International Hap MAP Project (HapMap Data Rel 24/phase II Nov08, on NCBI B36 assembly, dbSNPb126). All tagSNPs were included with a minor allele frequency (MAF) ≥ 0.05 and the criterion of linkage disequilibrium (LD) *r^2^* ≥0.8. Furthermore, a functional candidate strategy was also applied to select potential functional SNPs on the bioinformatics effect prediction website (SNPINFO, https://snpinfo.niehs.nih.gov/). Finally, three tagSNPs of *ACTB* gene, rs852423 (A > G), rs852426 (T > C), and rs2966449 (T > C), were selected and genotyped in this study. Detailed biological information was summarized in **Table S1**.

### Blood Samples Collecting and Genotyping

After an overnight fasting, blood samples were collected in vacuum anticoagulation tubes with ethylenediamine tetraacetic acid dipotassium salt (EDTA-K2)-containing for genotyping. DNA was extracted from the frozen whole blood by a protein precipitation method (Eaglink Cat EGEN2024, NANJING YININGFUSHENG Biotech. Co., Ltd. Nanjing, China) and stored at −20°C. The concentration and the purity of each DNA sample were determined using the NanoDrop 2000 spectrophotometer (Thermo Fisher Scientific, Waltham, MA). Amplifications of three SNPs were performed using a polymerase chain reaction (PCR)-TaqMan MGB probe array in the GeneAmp^®^ PCR system 9700 thermal cycler (Applied Biosystems, USA). The results were determined on the platform of 7900HT real-time PCR System (Applied Biosystems, Foster City, CA) with a Sequence Detection System (SDS) 2.3 software. To test the genetic effect of SNP, the code of wild types (WT) was 0 (reference), heterozygous types (HT) was 1, and the mutant types (MT) was 2 in additive model. Similar principle was employed in the dominant and recessive models. The successful call rates of SNPs genotyping were 100%. The genotype results were validated by Sanger sequencing, and the consistence was 100% (**Supplementary Materials**).

### Statistical Analysis

EpiData 3.0 software (The EpiData Association, Odense, Denmark) was performed for duplicate entry, and consistency check was also available. Quantitative variables were expressed as the mean ± standard deviation (SD), and the categorical variables were presented by their counts and proportions. A Fisher’s exact test was used to estimate whether the genotype frequencies in controls met the Hardy–Weinberg equilibrium (HWE). Chi-square (χ^2^) test was performed to display the frequencies and distributions of alleles. Binary logistic regression was applied to calculate the odds ratio (OR) and corresponding 95% confidence interval (CI) with adjustment for covariates, and evaluate the genetic effects of different models. All above statistical analyses were conducted on SPSS version 17.0 (SPSS, Inc, Chicago, IL). Stata SE version 12.0 (StataCorp LP, College Station, TX) was used to estimate heterogeneity between groups. Statistical significance was set at 0.05 (*P* ≤ 0.05).

## Results

### Demographic and Clinical Characteristics of DKD Cases and Controls

**Table 1** shows the detailed demographic characteristics of included 1,510 couple individuals. The age (mean ± SD) of 69.87 ± 8.61 years in DKD case group was definitely comparable with that of 69.50 ± 8.74 years in control group (*P* = 0.190). BMI and the proportions of smokers and drinkers in case and control groups were also evenly matched. Nevertheless, the percentage of female in case group was slightly higher than that in the comparison group. Subjects in DKD group had higher levels of systolic pressure (SBP), diastolic pressure (DBP), triglyceride (TG), total cholesterol (TC), low-density lipoprotein cholesterol (LDL-C), glucose (GLU), and shorter diabetic duration while lower mean high-density lipoprotein cholesterol (HDL-C) and eGFR than controls (all *P* values <0.05).

**Table 1:** Demographic and clinical characteristics of DKD cases and controls.

Characteristics	Group	Case *n* = 1,510	Control *n* = 1,510	*t*/χ^2^	*P*
Age (year)		69.87 ± 8.61	69.50 ± 8.74	1.186	0.190
Gender (%)	Male	543 (35.96%)	606 (40.13%)	7.576	0.018
	Female	967 (64.04%)	904 (59.87%)		
SB*P* (mm Hg)		153.78 ± 22.49	150.98 ± 19.98	3.603	<0.001
DB*P* (mm Hg)		79.47 ± 11.9	79.13 ± 10.4	0.820	<0.001
BMI (kg/m^2^)		25.02 ± 3.45	24.93 ± 3.52	0.661	0.258
TC (mmol/L)		5.41 ± 1.64	5.22 ± 1.12	3.788	<0.001
TG (mmol/L)		2.13 ± 1.64	1.86 ± 1.54	4.570	0.002
HDL-C (mmol/L)		1.50 ± 0.5	1.51 ± 0.4	0.299	<0.001
LDL-C (mmol/L)		3.26 ± 1.24	3.15 ± 0.92	2.953	<0.001
GLU (mmol/L)		9.68 ± 4.25	9.24 ± 3.05	3.222	<0.001
eGFR (ml·min^−1^·1.73 m^−2^)		58.69 ± 18.52	83.41 ± 13.44	41.986	<0.001
Smoking (%)	Yes	396 (26.23%)	404 (26.75%)	0.109	0.741
	No	1114 (73.77%)	1106 (73.25%)		
Drinking (%)	Yes	257 (17.02%)	306 (20.26%)	5.247	0.073
	No	1247 (82.58%)	1198 (79.34%)		
	Unknown	6 (0.40%)	6 (0.40%)		

SBP, systolic blood pressure; DBP, diastolic blood pressure; BMI, body mass index; TC, total cholesterol; TG, triglyceride; HDL-C, high-density lipoprotein cholesterol; LDL-C, low-density lipoprotein cholesterol; GLU, glucose; eGFR, estimated glomerular filtration rate.

### Association Analysis of the *ACTB* Polymorphisms with DKD

In this study, the genotype distributions of three tagSNPs exactly followed HWE in the control population (all *P* values > 0.05). We observed that there was difference of rs852423 genotypes between DKD cases and controls, after the adjustment for age, gender, smoking, drinking, BMI, diabetic duration, TG, TC, HDL-C, and LDL-C and the borderline *P* values were 0.046, 0.048, and 0.287 for additive, dominant, and recessive models, respectively (**Table 2**). rs852426 T > C variation presented a remarkable higher risk of DKD in the whole study population, the adjusted OR [95% confidence interval (CI)] of additive model was 1.217 (1.079–1.373) with a *P* value of 0.001. After Bonferonni correction (*P*×3), the association still remains statistically significant (*P* = 0.003). The association of rs2966449 (T > C) with DKD was also significant, and the adjusted OR (95% CI) of additive model was 1.151 (1.025–1.293) with a *P* value of 0.018. When rs852426 and rs2966449 entered the logistic regression model at the same time, the association of rs852426 still remained statistically significant (*P* = 0.016), whereas rs2966449 did not (*P* = 0.202).

**Table 2:** Association analysis of SNPs of the *ACTB* gene with DKD.

SNP	Group	WT/HT/MT	Genotype OR (95% CI) *P* value^a^			Allele gene		
			Additive model	Dominant model	Recessive model	Major/minor	OR (95%CI)/*P* ^b^	*P* ^c^
rs852423		AA/AG/GG				A/G		
	Case	716/648/146	1.12 (1.002–1.253)	1.157 (1.001–1.336)	1.147 (0.891–1.478)	2080/940	1.115 (0.999–1.245)	0.960
	Control	765/619/126	*P* = 0.046	*P* = 0.048	*P* = 0.287	2149/871	*P* = 0.053	
rs852426		TT/TC/CC				T/C		
	Case	890/518/102	1.217 (1.079–1.373)	1.226 (1.057–1.422)	1.522 (1.108–2.089)	2298/722	1.219 (1.079–1.376)	0.300
	Control	961/479/70	*P* = 0.001	*P* = 0.007	*P* = 0.009	2401/619	*P* = 0.001	
rs2966449		TT/TC/CC				T/C		
	Case	810/589/111	1.151 (1.025–1.293)	1.201 (1.039–1.389)	1.151 (0.865–1.533)	2289/811	1.107 (0.987–1.243)	0.307
	Control	874/540/96	*P* = 0.018	*P* = 0.013	*P* = 0.334	2288/732	*P* = 0.083	

WT, wild type; HT, heterozygote; MT, mutant type.

a Adjusted for age, gender, smoking, drinking, BMI and diabetic duration, TG, TC, HDL-C and LDL-C.

b P value of χ^2^ text for comparison of allele frequencies between case and control groups.

c P value of Fisher’s exact two-sided chi-squared test for Hardy–Weinberg equilibrium.

### Stratified Analyses by Age, Gender, Smoking, Drinking, and DM Duration

The ORs (95% CI) of rs852426 (TC + CC vs. TT) for DKD in 70 years or younger and older than 70 years populations were 1.058 (0.857–1.306) and 1.443 (1.168–1.782) with *P* values of 0.601 and 0.001, respectively. Remarkably, there was significant heterogeneity of the association between the two groups with *P* of 0.047 (**Table 3**).

**Table 3:** Stratified analysis of the *ACTB* gene with DKD by age.

SNP	Age	Group	WT/HT/MT	Genotype OR (95% CI)^a^					
				Additive model		Dominant model		Recessive model	
			AA/AG/GG		Heterogeneity test		Heterogeneity test		Heterogeneity test
rs852423	≤70 years	Case	338/305/70	1.124 (0.959–1.318)	*P* = 0.994	1.147 (0.933–1.41)	*P* = 0.912	1.197 (0.837–1.712)	*P* = 0.854
		Control	387/324/66	*P* = 0.150		*P* = 0.193		*P* = 0.324	
	>70 years	Case	378/343/76	1.125 (0.960–1.317)		1.166 (0.951–1.429)		1.140 (0.794–1.637)	
		Control	378/295/60	*P* = 0.145		*P* = 0.139		*P* = 0.477	
			TT/TC/CC		Heterogeneity test		Heterogeneity test		Heterogeneity test
rs852426	≤70 years	Case	426/240/47	1.130 (0.949–1.344)	*P* = 0.208	1.058 (0.857–1.306)	*P* = 0.047	1.815 (1.132–2.908)	*P* = 0.375
		Control	472/274/31	*P* = 0.170		*P* = 0.601		*P* = 0.013	
	>70 years	Case	464/278/55	1.323 (1.116–1.568)		1.443 (1.168–1.782)		1.332 (0.866–2.048)	
		Control	489/205/39	*P* = 0.001		*P* = 0.001		*P* = 0.192	
			TT/TC/CC		Heterogeneity test		Heterogeneity test		Heterogeneity test
rs2966449	≤70 years	Case	386/270/57	1.143 (0.969–1.35)	*P* = 0.834	1.121 (0.911–1.380)	*P* = 0.336	1.446 (0.960–2.178)	*P* = 0.190
		Control	4336/296/45	*P* = 0.113		*P* = 0.280		*P* = 0.078	
	>70 years	Case	424/319/54	1.172 (0.994–1.381)		1.296 (1.055–1.592)		0.959 (0.640–1.436)	
		Control	438/244/51	*P* = 0.059		*P* = 0.013		*P* = 0.838	

a Adjusted for gender, smoking, drinking, BMI and diabetic duration, TG, TC, HDL-C and LDL-C.

Also, a significant association of rs852426 and DKD was found in populations of male and female, without smoking and without drinking and with DM duration of 10–20 years (**Tables S2–5**). However, no significant heterogeneity was observed between different strata of gender, smoking, drinking, and DM duration.

In addition, the variation of rs2966449 showed significant association with DKD in the populations of older than 70 years, male, without smoking, and without drinking and with DM duration over 20 years (**Tables S2–5**). No significant heterogeneity was observed between different strata of gender, smoking, drinking, and DM duration either.

### Stratified Analyses by DKD Severity

Further, we divided the DKD cases by its severity with an eGFR of 60 ml·min^−1^·1.73 m^−2^, and the same control group was used to explore the effect of DKD severity on the associations of SNPs and DKD susceptibility (**Table S6**). The results indicated that rs852426 was significantly associated with DKD, the ORs (95% CI) and corresponding *P* values were 1.231 (1.047–1.448) (*P* = 0.012) and 1.181 (1.027–1.357) (*P* = 0.019) in additive model.

### Analyses for SNPs of the *ACTB* Polymorphisms and eGFR

One-way ANOVA revealed that the eGFR levels of the individuals with TT and CC genotypes of rs2966449 were significantly lower than that of TC genotype in DKD cases (*P* = 0.021). Even adjusting for gender, smoking, drinking, BMI, and diabetic duration, the difference still remained (*P* = 0.013), whereas no significant difference of eGFR was found among the genotypes of rs852423 and rs852426 (**Table 4**).

**Table 4:** Comparison of eGFR among the genotypes of *ACTB*.

SNP	DKD Group	eGFR (mean ± SD)			*P*
rs852423		AA	AG	GG	
	Case	57.97 ± 18.26	59.67 ± 18.74	57.83 ± 18.74	0.203
	Control	83.02 ± 13.58	84.17 ± 13.37	82.11 ± 12.83	0.152
rs852426		TT	TC	CC	
	Case	58.46 ± 18.68	59.06 ± 18.26	58.74 ± 18.57	0.840
	Control	83.01 ± 13.82	84.34 ± 12.78	82.67 ± 12.51	0.189
rs2966449		TT	TC	CC	
	Case	57.88 ± 18.46	60.26 ± 18.53	56.23 ± 18.47	0.021^a^
	Control	83.02 ± 13.35	84.39 ± 13.62	81.55 ± 13.04	0.065

a The average eGFR levels of the TT and CC genotypes of rs2966449 was significantly lower than that of TC genotype in DKD cases (P < 0.05 by SNK test).

## Discussion

Although the GWAS have identified several DN associated loci for European and African populations (Pezzolesi and Krolewski, 2013; Raina et al., 2015), only few of them have been validated, and many remained unvalidated (Regele et al., 2015). Previous GWAS identified 16 SNPs accounted for only 1.4% of the variability of eGFR (Kottgen et al., 2009). Besides, the functional effect of genetic loci often differs across different ethnicities and populations. More promising candidate genes for DKD remain to be explored.

Because *ACTB* loss-of-function mutations result in renal agenesis and impairment, the study of the common variants at *ACTB* and CKD as well as eGFR would be deserved. In our present study, rs852426 T to C variation, located at the upstream of *ACTB*, is significantly associated with DKD, and the association is particularly significant in the older than 70 years populations with even higher OR (95% CI) of 1.425 (1.155–1.759). The results suggest that aging might promote the impact of rs852426 T to C variation on the development of DKD. We also observed that individuals with TT or CC genotypes of rs2966449 presented lower eGFR level than those with TC genotype, and the result is consistent with the functional characteristics that *ACTB* haploinsufficiency leads to reduced cell proliferation, altered expression of cell-cycle genes, and decreased amounts of nuclear (Cuvertino et al., 2017). Previous studies found that DKD occurs incrementally during T2DM in those with long diabetic duration or advanced age (Satirapoj and Adler, 2014). Thus, this study would provide an available genetic marker of precisive prevention from DKD for T2DM patients.

Obviously, the average age in this study, which is near 70 years, has efficient power to find the cut-point of age heterogeneity of the association. The association of rs852426 and DKD also emerged in the subgroup without smoking and drinking and with DM duration of 10–20 years. So, further replication study would warrant large sample to identify the target population of genetic susceptibility to the variation of rs852426. The weak association of rs2966449 and DKD observed in this study is necessary to be replicated by further study.

Previous study demonstrated that β-cytoplasmic actin structures were disorganized, and its expression was downregulated in the epithelial-to-mesenchymal transition (EMT) process in the involvement of fibroblastic phenotype (Shagieva et al., 2012). Ishizawa et al. (2014) reported that Rho kinase was stimulated by its upstream factors in cultured renal tubular cells, triggering EMT to develop to renal fibrosis. In addition, the *ACTB* gene is a downstream gene in the Rho/ROCK pathway. Besides, animal experiment has reported that neonatal rats subjected to partial unilateral ureteral obstruction (PUUO) could produce more actin filaments to control and maintain kidney shape and internal structure (Zhao et al., 2016). Along with the evidence mentioned above, we therefore speculated with caution that the *ACTB* gene may take a role in the EMT-induced DKD. Bioinformatic analysis indicated that the variations of three tagging SNPs selected in this study as well as other SNPs with MAF >0.05 in *ACTB* would not cause the structural changes of amino acids; we speculate that the variations of rs852426 and rs2966449 might affect the transcriptional level of *ACTB* by an epigenetic modification way. Further biological functional research would be warranted to explore the potential effect of the variants at *ACTB* on gene transcription, splicing, or mRNA stability as well as the molecular mechanism of DKD.

Our study for the first time explored the associations of the *ACTB* genetic variations with DKD and age- and gender-matched comparison, which was adopted to balance the common confounding factors. Besides, a standard that subjects with T2DM duration more than 5 years were qualified for the study was applied to weaken the discrimination from diabetic duration, which was also guaranteed by statistical adjustments. Moreover, the current subjects were representative because they were selected from about 20,340 T2DM patients from community-based chronic disease management system, distributed in the southern and northern of Jiangsu Province.

There indeed consisted several limitations that should not be ignored. First, we selected candidate SNPs at the *ACTB* with the criterion of MAF ≥ 0.05 and could have missed the rare variants with MAF <0.05 that may have substantial effects on the occurrence and progress of DKD. Second, case-control design cannot avoid the causal confusion caused by time sequence. Besides, the diabetic duration in this study referred to the time gap from first-diagnosed T2DM to the investigation day, which may overestimate the onset time of DKD. Further follow-up study is warranted.

## Conclusion

Data from the present study suggest that the *ACTB* genetic variants confer the susceptibility to DKD. SNP rs852426 at this gene is associated with the increased risk of DKD while the association is particularly marked in T2DM patients over 70 years old. SNP rs2966449, however, shows a mild association with DKD and eGFR.

## Ethics Statement

Informed consent was obtained from all participants and approved by the Research Ethics Committee of Jiangsu Provincial Center for Disease Control and Prevention and Nanjing Medical University.

## Author Contributions

CS and MW designed the program. MW, YQ, JZ, JS, EP, QZ, NZ, and HS collected the data. ML, JD, and YT extracted DNA and performed genotyping work. ML analyzed the data and wrote the manuscript. CS edited and proofed the manuscript.

## Conflict of Interest Statement

The authors declare that the research was conducted in the absence of any commercial or financial relationships that could be construed as a potential conflict of interest.
