# Supplementary material for: ACTB Variants Confer the Genetic Susceptibility to Diabetic Kidney Disease in a Han Chinese Population
Source: Front Genet. 2019 Jul 23;10:663. doi: 10.3389/fgene.2019.00663 (PMC6664243; doi:10.3389/fgene.2019.00663)
Supplement: Supplementary file 1 [file Table_1.docx]

**Supplementary tables**

| Table S1. The biological information and function prediction of three tagSNPs of the *ACTB* gene. | | | | | | | | | | | |
| --- | --- | --- | --- | --- | --- | --- | --- | --- | --- | --- | --- |
| No | SNP | Chromosome | Position | Allele | LDsnp | Pop/LD | TFBS | RegPotential | Conservation | Nearby Gene | Distance (bp) |
| 1 | rs852423 | 7 | 5534892 | A/G | rs852423 | 1 | -- | 0.265805 | 0 | ACTB | 1580\|\|1855 |
| 2 | rs852426 | 7 | 5532879 | T/C | rs852426 | 1 | -- | 0 | 0.002 | MIRN589\|\|ACTB | -30806\|\|-433 |
| 3 | rs2966449 | 7 | 5538151 | T/C | rs2966449 | 1 | Msx-1 | 0.13539 | 0 | ACTB\|\|FSCN1 | -1404\|\|-60829 |

| Table S2. Stratified analysis of the *ACTB* gene with DKD by gender | | | | | | | | | |
| --- | --- | --- | --- | --- | --- | --- | --- | --- | --- |
| SNP | Gender | Group | WT/HT/MT | Genotype OR (95% CI) ^a^ | | | | |  |
|  |  |  |  | Additive model | | Dominant model |  | Recessive model |  |
| rs852423 |  |  | AA/AG/GG |  | Heterogeneity test |  | Heterogeneity test |  | Heterogeneity test |
|  | Male | Case | 257/234/52 | 1.166(0.971-1.401) | *P*=0.594 | 1.164(0.921-1.472) | *P*=0.973 | 1.381(0.905-2.107) | *P*=0.295 |
|  |  | Control | 308/254/44 | *P*=0.100 |  | *P*=0.203 |  | *P*=0.135 |  |
|  | Female | Case | 459/424/94 | 1.094(0.950-1.259) |  | 1.158(0.963-1.392) |  | 1.015(0.739-1.395) |  |
|  |  | Control | 457/365/82 | *P*=0.215 |  | *P*=0.118 |  | *P*=0.925 |  |
| rs852426 |  |  | TT/TC/CC |  | Heterogeneity test |  | Heterogeneity test |  | Heterogeneity test |
|  | Male | Case | 320/179/44 | 1.203(0.994-1.455) | *P*=0.906 | 1.170(0.921-1.487) | *P*=0.635 | 1.710(1.057-2.767) | *P*=0.504 |
|  |  | Control | 380/196/30 | *P*=0.057 |  | *P*=0.198 |  | *P*=0.029 |  |
|  | Female | Case | 570/339/58 | 1.221(1.044-1.429) |  | 1.260(1.042-1.524) |  | 1.356(0.889-2.068) |  |
|  |  | Control | 581/283/40 | *P*=0.013 |  | *P*=0.017 |  | *P*=0.158 |  |
| rs2966449 |  |  | TT/TC/CC |  | Heterogeneity test |  | Heterogeneity test |  | Heterogeneity test |
|  | Male | Case | 293/208/42 | 1.221(1.009-1.477) | *P*=0.472 | 1.222(0.965-1.547) | *P*=0.902 | 1.556(0.961-2.52) | *P*=0.183 |
|  |  | Control | 356/219/31 | *P*=0.040 |  | *P*=0.097 |  | *P*=0.072 |  |
|  | Female | Case | 517/381/69 | 1.116(0.964-1.293) |  | 1.199(0.996-1.443) |  | 0.974(0.681-1.393) |  |
|  |  | Control | 518/321/65 | *P*=0.143 |  | *P*=0.055 |  | *P*=0.885 |  |

a, adjusted for gender, smoking, drinking, BMI, diabetic duration, TG, TC, HDL-C and LDL-C.

Table S3. Stratified analysis of the *ACTB* gene with DKD by smoking status

| SNP | Smoking | Group | WT/HT/MT | OR (95% CI) ^a^ | | | | |  |
| --- | --- | --- | --- | --- | --- | --- | --- | --- | --- |
|  |  |  |  | Additive model | | Dominant model |  | Recessive model |  |
| rs852423 |  |  | AA/AG/GG |  | Heterogeneity test |  | Heterogeneity test |  | Heterogeneity test |
|  | Yes | Case | 186/169/41 | 1.075(0.865-1.336) | *P*=0.658 | 1.063(0.801-1.411) | *P*=0.489 | 1.200(0.739-1.947) | *P*=0.848 |
|  |  | Control | 119/176/35 | *P*=0.512 |  | *P*=0.672 |  | *P*=0.462 |  |
|  | No | Case | 530/479/105 | 1.138(0.999-1.297) |  | 1.192(1.008-1.411) |  | 1.132(0.841-1.524) |  |
|  |  | Control | 572/443/91 | *P*=0.052 |  | *P*=0.040 |  | *P*=0.415 |  |
| rs852426 |  |  | TT/TC/CC |  | Heterogeneity test |  | Heterogeneity test |  | Heterogeneity test |
|  | Yes | Case | 242/127/27 | 1.089(0.860-1.378) | *P*=0.289 | 1.022(0.765-1.366) | *P*=0.150 | 1.623(0.870-3.030) | *P*=0.818 |
|  |  | Control | 250/136/18 | *P*=0.479 |  | *P*=0.883 |  | *P*=0.128 |  |
|  | No | Case | 648/391/75 | 1.259(1.093-1.45) |  | 1.298(1.091-1.543) |  | 1.480(1.023-2.141) |  |
|  |  | Control | 711/343/52 | *P*=0.001 |  | *P*=0.003 |  | *P*=0.037 |  |
| rs2966449 |  |  | TT/TC/CC |  | Heterogeneity test |  | Heterogeneity test |  | Heterogeneity test |
|  | Yes | Case | 207/159/30 | 1.211(0.961-1.525) | *P*=0.652 | 1.238(0.931-1.645) | *P*=0.845 | 1.387(0.775-2.480) | *P*=0.532 |
|  |  | Control | 230/152/22 | *P*=0.104 |  | *P*=0.142 |  | *P*=0.270 |  |
|  | No | Case | 603/430/82 | 1.137(0.993-1.301) |  | 1.197(1.010-1.418) |  | 1.091(0.785-1.518) |  |
|  |  | Control | 644/388/74 | *P*=0.063 |  | *P*=0.038 |  | *P*=0.604 |  |

a, adjusted for age, gender, drinking, BMI, diabetic duration, TG, TC, HDL-C and LDL-C.

| Table S4. Stratified analysis of the *ACTB* gene with DKD by drinking status | | | | | | | | | |
| --- | --- | --- | --- | --- | --- | --- | --- | --- | --- |
| SNP | Drinking | Group | WT/HT/MT | Genotype OR (95% CI) ^a^ | | | | |  |
|  |  |  |  | Additive model | | Dominant model |  | Recessive model |  |
| rs852423 |  |  | AA/AG/GG |  | Heterogeneity test |  | Heterogeneity test |  | Heterogeneity test |
|  | Yes | Case | 124/114/19 | 1.062(0.813-1.386) | *P*=0.672 | 1.161(0.825-1.636) | *P*=0.958 | 0.848(0.457-1.572) | *P*=0.259 |
|  |  | Control | 155/123/28 | *P*=0.661 |  | *P*=0.392 |  | *P*=0.601 |  |
|  | No | Case | 590/532/125 | 1.131(0.999-1.280) |  | 1.149(0.979-1.349) |  | 1.227(0.926-1.626) |  |
|  |  | Control | 607/494/97 | *P*=0.053 |  | *P*=0.090 |  | *P*=0.155 |  |
| rs852426 |  |  | TT/TC/CC |  | Heterogeneity test |  | Heterogeneity test |  | Heterogeneity test |
|  | Yes | Case | 155/87/15 | 1.061(0.802-1.404) | *P*=0.293 | 1.104(0.780-1.562) | *P*=0.538 | 0.971(0.474-1.988) | *P*=0.153 |
|  |  | Control | 192/96/18 | *P*=0.678 |  | *P*=0.578 |  | *P*=0.935 |  |
|  | No | Case | 733/427/87 | 1.246(1.089-1.426) |  | 1.243(1.054-1.466) |  | 1.682(1.176-2.406) |  |
|  |  | Control | 766/380/52 | *P*=0.001 |  | *P*=0.010 |  | *P*=0.004 |  |
| rs2966449 |  |  | TT/TC/CC |  | Heterogeneity test |  | Heterogeneity test |  | Heterogeneity test |
|  | Yes | Case | 142/98/17 | 1.082(0.819-1.429) | *P*=0.629 | 1.055(0.749-1.484) | *P*=0.399 | 1.327(0.653-2.697) | *P*=0.718 |
|  |  | Control | 171/118/17 | *P*=0.579 |  | *P*=0.760 |  | *P*=0.434 |  |
|  | No | Case | 666/489/92 | 1.166(1.025-1.327) |  | 1.235(1.050-1.451) |  | 1.127(0.822-1.545) |  |
|  |  | Control | 700/420/78 | *P*=0.019 |  | *P*=0.011 |  | *P*=0.459 |  |

a, adjusted for age, gender, smoking, BMI, diabetic duration, TG, TC, HDL-C and LDL-C.

| Table S5. Stratified analysis of the *ACTB* gene with DKD by DM Duration | | | | | | | | | |
| --- | --- | --- | --- | --- | --- | --- | --- | --- | --- |
| SNP | DM Duration | Group | WT/HT/MT | Genotype OR (95% CI) ^a^ | | | | |  |
|  |  |  |  | Additive model | | Dominant model |  | Recessive model |  |
| rs852423 |  |  | AA/AG/GG |  | Heterogeneity test | | Heterogeneity test | | Heterogeneity test |
|  | ≤10years | Case | 331/279/59 | 0.972(0.812-1.163) | *P*=0.141 | 1.017(0.806-1.284) | *P*=0.346 | 0.819(0.545-1.23) | *P*=0.167 |
|  |  | Control | 267/211/50 | *P*=0.755 |  | *P*=0.886 |  | *P*=0.335 |  |
|  | 10-20years | Case | 322/288/71 | 1.172(1.001-1.373) |  | 1.181(0.962-1.405) |  | 1.358(0.950-1.941) |  |
|  |  | Control | 432/345/66 | *P*=0.049 |  | *P*=0.112 |  | *P*=0.093 |  |
|  | >20years | Case | 63/81/16 | 1.439(0.977-2.118) |  | 1.547(0.956-2.504) |  | 1.584(0.647-3.876) |  |
|  |  | Control | 66/63/10 | *P*=0.065 |  | *P*=0.076 |  | *P*=0.314 |  |
| rs852426 |  |  | TT/TC/CC |  | Heterogeneity test | | Heterogeneity test | | Heterogeneity test |
|  | ≤10years | Case | 395/230/44 | 1.120(0.923-1.358) | *P*=0.401 | 1.145(0.902-1.453) | *P*=0.301 | 1.176(0.717-1.927) | *P*=0.594 |
|  |  | Control | 327/172/29 | *P*=0.251 |  | *P*=0.266 |  | *P*=0.521 |  |
|  | 10-20years | Case | 395/242/44 | 1.325(1.114-1.576) |  | 1.379(1.116-1.703) |  | 1.584(0.999-2.510) |  |
|  |  | Control | 551/256/36 | *P*=0.001 |  | *P*=0.003 |  | *P*=0.050 |  |
|  | >20years | Case | 100/46/14 | 1.103(0.745-1.633) |  | 0.970(0.596-1.580) |  | 2.268(0.757-6.790) |  |
|  |  | Control | 83/51/5 | *P*=0.626 |  | *P*=0.904 |  | *P*=0.143 |  |
| rs2966449 |  |  | TT/TC/CC |  | Heterogeneity test | | Heterogeneity test | | Heterogeneity test |
|  | ≤10years | Case | 367/255/47 | 1.047(0.868-1.263) | *P*=0.239 | 1.088(0.861-1.376) | *P*=0.365 | 0.950(0.598-1.509) | *P*=0.507 |
|  |  | Control | 304/188/36 | *P*=0.633 |  | *P*=0.479 |  | *P*=0.829 |  |
|  | 10-20years | Case | 368/263/50 | 1.168(0.990-1.378) |  | 1.205(0.980-1.481) |  | 1.247(0.830-1.875) |  |
|  |  | Control | 492/299/52 | *P*=0.066 |  | *P*=0.078 |  | *P*=0.288 |  |
|  | >20years | Case | 75/71/14 | 1.604(1.080-2.381) |  | 1.720(1.059-2.791) |  | 2.104(0.777-5.700) |  |
|  |  | Control | 78/53/8 | *P*=0.019 |  | *P*=0.028 |  | *P*=0.143 |  |

a, adjusted for age, gender, smoking, drinking, BMI, TG, TC, HDL-C and LDL-C.

Table S6. Stratified analysis of the *ACTB* gene with DKD by DKD severity

| DKD severity | SNP | Genotype OR (95%CI) *P* value ^a^ | | |
| --- | --- | --- | --- | --- |
|  |  | Additive model | Dominant model | Recessive model |
| eGFR<60 | rs852423 | 1.103(0.970-1.254) | 1.121(0.949-1.325) | 1.167(0.873-1.559) |
|  |  | *P*=0.134 | *P*=0.179 | *P*=0.296 |
|  | rs852426 | 1.205(1.048-1.384) | 1.199(1.010-1.423) | 1.562(1.094-2.231) |
|  |  | *P*=0.009 | *P*=0.038 | *P*=0.014 |
|  | rs2966449 | 1.135(0.994-1.296) | 1.146(0.968-1.355) | 1.279(0.928-1.763) |
|  |  | *P*=0.061 | *P*=0.113 | *P*=0.133 |
| eGFR≥60 | rs852423 | 1.143(0.985-1.328) | 1.218(1.003-1.480) | 1.089(0.775-1.529) |
|  |  | *P*=0.079 | *P*=0.047 | *P*=0.624 |
|  | rs852426 | 1.252(1.065-1.472) | 1.287(1.056-1.569) | 1.465(0.963-2.229) |
|  |  | *P*=0.006 | *P*=0.012 | *P*=0.075 |
|  | rs2966449 | 1.169(1-1.367) | 1.288(1.060-1.565) | 0.953(0.637-1.425) |
|  |  | *P*=0.049 | *P*=0.011 | *P*=0.815 |

a, adjusted for age, gender, smoking, drinking, BMI, diabetic duration, TG, TC, HDL-C and LDL-C.
